# Island-wide characterization of agricultural production challenges the demographic collapse hypothesis for Rapa Nui (Easter Island)

**DOI:** 10.1126/sciadv.ado1459

**Published:** 2024-06-21

**Authors:** Dylan S. Davis, Robert J. DiNapoli, Gina Pakarati, Terry L. Hunt, Carl P. Lipo

**Affiliations:** ^1^ Columbia Climate School, Columbia University, New York, NY USA.; ^2^ Division of Biology and Paleoenvironment, Lamont-Doherty Earth Observatory, Palisades, NY USA.; ^3^ Columbia Center for Archaeology, Columbia University, New York, NY USA.; ^4^ Office of Strategic Research Initiatives, Binghamton University, Binghamton, NY USA.; ^5^ Environmental Studies Program, Binghamton University, Binghamton, NY USA.; ^6^ Independent Researcher, Rapa Nui, Chile.; ^7^ School of Anthropology, University of Arizona, Tuscon, AZ USA.; ^8^ Department of Anthropology, Binghamton University, Binghamton, NY USA.

## Abstract

Communities in resource-poor areas face health, food production, sustainability, and overall survival challenges. Consequently, they are commonly featured in global debates surrounding societal collapse. Rapa Nui (Easter Island) is often used as an example of how overexploitation of limited resources resulted in a catastrophic population collapse. A vital component of this narrative is that the rapid rise and fall of pre-contact Rapanui population growth rates was driven by the construction and overexploitation of once extensive rock gardens. However, the extent of island-wide rock gardening, while key for understanding food systems and demography, must be better understood. Here, we use shortwave infrared (SWIR) satellite imagery and machine learning to generate an island-wide estimate of rock gardening and reevaluate previous population size models for Rapa Nui. We show that the extent of this agricultural infrastructure is substantially less than previously claimed and likely could not have supported the large population sizes that have been assumed.

## Teaser

Satellite image analyses drastically reduce rock gardening estimates on Easter Island, indicating a small pre-European population.

## INTRODUCTION

**Fig. 1.**
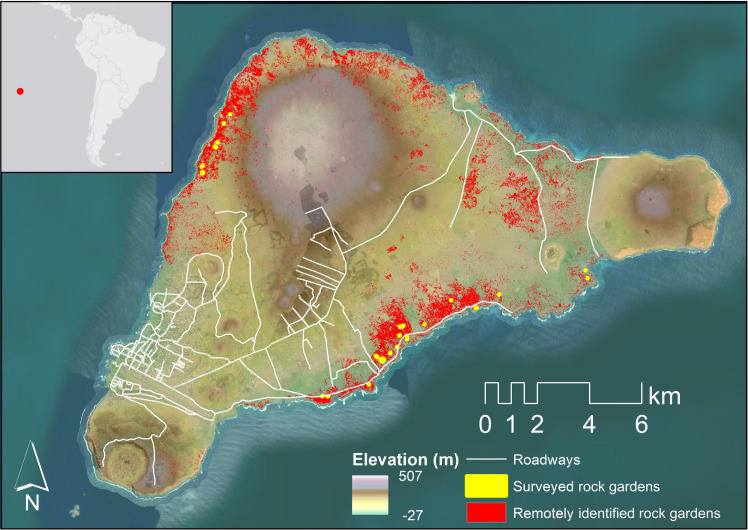
Map of Rapa Nui and its location in the southeastern Pacific. Satellite data provided by Maxar. Service layer credits: NASA Shuttle Radar Topography Mission (*79*).

Rapa Nui is one of Earth’s most remote human-populated locations (Fig. 1). It is over 2000 km from the nearest inhabited island (Pitcairn) and over 3700 km from the South American mainland. The island is small (~164 km^2^) and has relatively limited soil productivity and freshwater sources (*1*–*8*). The physical constraints of the island limited opportunities for cultivation practices such as terraced irrigation systems found elsewhere in Polynesia. Instead, past Rapanui communities initially mitigated problems of the island’s poor soil productivity by burning the native palm vegetation (*9*), a practice common in swidden cultivation. Over time, however, local communities also began to increasingly engage in a cultivation strategy known as “lithic mulching,” a form of rock gardening (*1*, *4*, *10*–*14*). These rock gardens enhanced plant productivity by increasing available soil nutrients and maintaining soil moisture (*15*). Yet, our understanding of the extent of rock gardening practices on Rapa Nui is limited, and current estimates are inaccurate (see more below). Here, we present an approach for mapping rock gardening features using high-resolution short-wave infrared (SWIR) satellite imagery and machine learning.

**Fig. 2.**
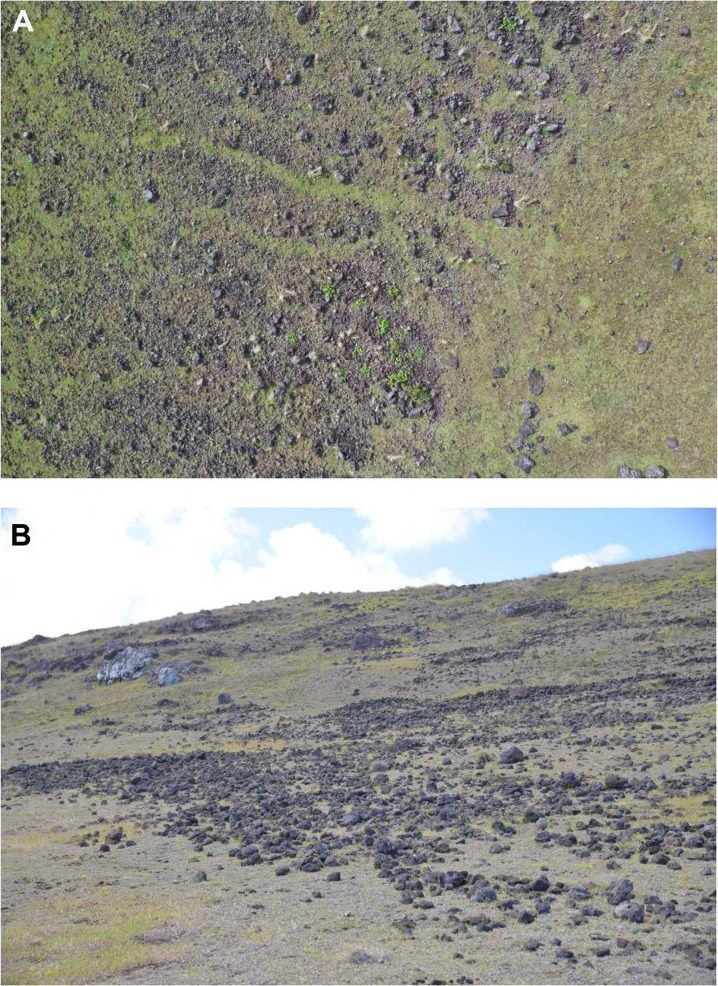
Rock gardening on Rapa Nui. (**A**) View from a low-altitude drone. (**B**) View from the ground.

Rock gardening (sometimes referred to as lithic mulching) is a term for adding rocks to cultivation areas (Fig. 2). The practice of rock gardening can be found worldwide (*16*–*19*). On Rapa Nui, rock gardening practices take three different forms (*15*). First, “veneer gardens” comprise a layer of fist-sized rocks placed directly on the surface. Second, lithic mulching adds broken rock material into the first 20 to 25 cm of soil. Third, boulder gardens include additional large stones on the surface. These forms can appear as a continuum with varying-sized rocks and a variable abundance of added material.

Overall, rock gardening increases productivity in a variety of ways (*20*). First, placing rocks on the surface can protect plants by generating more turbulent airflow over the garden surface. In this way, rock gardening can reduce the highest daytime temperatures and increase the lowest nighttime temperatures. Adding a layer of rocks to a garden mediates temperature swings, producing a more stable environment for plant growth (*21*). Second, the disrupted airflow also limits the wind, which can desiccate foliage while providing shade to reduce soil moisture evaporation. Third, by mediating the climate, rock gardening can contribute to the enhancement of nutrient-poor soils by reducing nutrient leaching (*15*, *22*). The soils of Rapa Nui are volcanic in origin but highly weathered. Consequently, they are relatively depleted in nutrients that are essential for plant growth, particularly nitrogen (N), phosphorus (P), and potassium (K), but also calcium (Ca), magnesium (Mg), and sulfur (S). As soil weathers, the availability of these nutrients—particularly N, P, and K—declines from soil moisture loss and rainfall-induced nutrient leaching of soils (*4*, *11*, *23*). Abundant rain can exacerbate the situation, as the greater the rainfall, the quicker the depletion of mineral nutrients. The placement of freshly broken rocks can increase the productivity of the soil by exposing unweathered surfaces and sources of minerals. Given its high surface-to-volume ratio, Stevenson *et al.* (*15*) suggest that pulverized rock has the most potential to add nutrients. Pulverized rock might accumulate as byproducts of digging pits into rock-strewn surfaces or from debris collected at quarrying areas.

Given the benefits rock gardening has for increasing soil productivity and, thus, plant growth, its practice was a vital part of pre-contact Rapanui subsistence (*1*, *14*, *15*, *21*). Nearly half of the Rapanui diet consisted of terrestrial foods (*24*, *25*). In this regard, measuring the extent of rock gardens is critical for understanding the island’s pre-contact environmental carrying capacity. To date, the only island-wide estimate for ancient rock gardening activity comes from the work of Ladefoged *et al.* (*26*). Using near-infrared (NIR) bands from Worldview-2 satellite images, they estimated that between 4.9 and 21.1 km^2^ of the island’s total 163.6 km^2^ area was covered by rock gardens. However, as Ladefoged *et al.* (*26*) report, the accuracy of this estimation is low (Table 1). Given that this dataset provides one of the critical parameters subsequently used to model population sizes on Rapa Nui (*6*), a re-assessment of the prevalence of rock gardening activity is required.

**Table 1.** Accuracy and performance metrics from Ladefoged *et al*. (*26*).

Model	Recall	Precision	F1	Overall accuracy	Kappa coefficient
MLC (maximal)	0.337	0.671	0.449	0.588	0.17
MLC (medial)	0.327	0.687	0.443	0.591	0.18
MLC (minimal)	0.319	0.753	0.448	0.609	0.22

In this paper, we reevaluate the extent of ancient rock gardening on Rapa Nui using new satellite imagery, including SWIR, near-infrared (NIR), and other visible spectra. The SWIR range is sensitive to water and nitrogen levels, which helps it distinguish between different kinds of vegetation and soil compositions resulting from different absorption characteristics of minerals (*27*–*30*). Human impacts on soil properties and vegetation are thus likely to be highlighted by SWIR due to its capacity to differentiate between subtle differences in environmental conditions. Despite this fact, SWIR data have historically been available only in low resolution (~20 to 60 m) and have limited use cases in archaeology [but see (*31*–*35*)]. Thus, these data offer great potential to improve our knowledge of rock gardening on Rapa Nui and, by extension, our understanding of pre-European contact population sizes. This study uses archaeological field identifications of rock garden features to train machine learning models to analyze island-wide SWIR data from the Worldview-3 satellite. Our findings indicate that the prevalence of rock gardening is approximately one-fifth of the most conservative previous estimates (*26*) that Puleston *et al.* (*6*) used to estimate population sizes. This result challenges the arguments of several scholars for a larger terrestrial carrying capacity. As such, we use these new estimates of rock gardening infrastructure to reevaluate previous population size models for Rapa Nui [e.g., (*9*)]. We demonstrate that the extent of rock gardening cultivation found in the occupied coastal areas comports with estimates of the population from observations made by early European visitors to the island in the 18th century, which is about 3000 (*36*).

### Previous approaches to identifying rock garden features using remote sensing

While many rock garden features have been recorded across the island, a systematic surface survey of rock gardening features still needs to be completed. To date, the only attempt to rectify this issue comes from a comprehensive island-wide estimate of rock gardens produced from a remote sensing survey conducted by Ladefoged *et al.* (*26*). Using high-resolution visual and near-infrared (VNIR) imagery from WorldView-2 and a maximum likelihood classifier (MLC), the authors estimate that lithic mulching fields and gardens cover between 4.9 and 21.1 km^2^ of the island. This work has provided a systematic island-wide assessment of rock gardening. These datasets, however, attained low rates of precision, recall, and overall accuracy (Table 1).

**Fig. 3.**
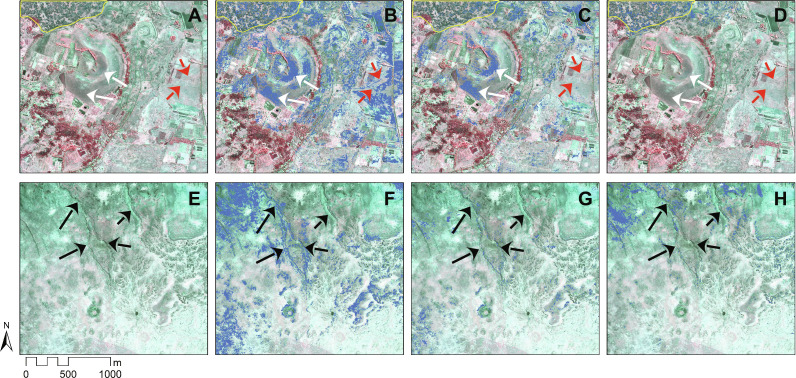
Classifications of landscape features in Ladefoged *et al.* (*26*) compared with the results of this study. (**A** and **E**) SWIR images of two regions of Rapa Nui, one near the north coast (A) and one near the west coast (E). Rock gardens are outlined in yellow, and nongarden features are indicated by arrows, including agricultural fields (white arrows), colluvial deposits (black arrows), and modern roadways (red arrows). (**B** to **D** and **F** to **H**) Comparison of Ladefoged *et al.* (*26*) maximal probability (B and F) and minimal probability (C and G) estimates and this study’s (D and H) estimates for rock gardening (in blue).

Issues of low accuracy warrant the creation of new analyses that can improve estimates of rock gardening. Improving these estimates is especially important because these data have been used to estimate population sizes at different points in the island’s human history as part of the ongoing debate over whether Rapa Nui represents an ecological success story or an example of a Malthusian trap where population exceeded the carrying capacity and fell into social and ecological collapse (*6*, *37*, *38*). Accurately determining the extent of rock gardening is one essential component in calculating likely population sizes and the island’s carrying capacity, along with better estimates of nonrock garden soil productivity and the longevity of other cultivation strategies on the island. Errors are present in existing estimates: The data produced by Ladefoged *et al.* (*26*) present numerous cases where areas identified as rock gardens are modern roads, natural lava flows, and vegetation (Fig. 3). Thus, their analysis likely greatly overestimates the amount of ancient rock gardening on Rapa Nui.

The misidentifications in Ladefoged *et al.*’s (*26*) analysis are likely due, in part, to the imagery data available at the time from the Worldview-2 satellite. These images provide nine bands of spectral data with wavelengths between 0.45 to 1.04 μm. The NIR bands (0.77 to 1.49 μm) are of particular value for measuring cultivation features as they tend to reflect differences in light absorption due to varying abundance of water. These bands, however, cover a relatively narrow range of the infrared spectrum (i.e., between 0.7 and 1000 μm). Expanding the spectral range to larger wavelengths can significantly increase the ability, for example, to discriminate between mineralogical and geological materials.

## RESULTS

We trained a series of machine learning models to identify rock gardening features in Worldview-3 imagery using data collected during ground surveys over the past 5 years. The results of our machine learning analyses are reported in Table 2. In all cases, SWIR data perform better than VNIR bands at identifying rock gardens and classifying the landscape of Rapa Nui. Combining VNIR with SWIR did not improve results. This finding suggests that the visual and NIR spectra lack the spectral discriminatory power to distinguish between rock gardens and other rocky landscape components on Rapa Nui.

**Table 2.** Results of machine learning classifications tested using VNIR and SWIR imagery from Worldview-3. SWIR outperformed all VNIR applications and performed best using a maximum entropy classifier.

Imagery type	Classifier	Recall	Precision	F1	Overall accuracy	Kappa coefficient
VNIR	Random forest	0.716	0.740	0.728	0.7091	0.6505
VNIR	Maximum entropy	0.701	0.697	0.699	0.695	0.6342
VNIR	Maximum likelihood	0.592	0.619	0.605	0.584	0.5002
SWIR	Random forest	0.753	0.715	0.734	0.7485	0.6915
SWIR	Maximum entropy	0.834	0.827	0.830	0.8244	0.7865
SWIR	Maximum likelihood	0.743	0.744	0.743	0.7765	0.7248
VNIR + SWIR	Maximum entropy	0.779	0.799	0.789	0.7817	0.7378

The results of the maximum entropy model applied to the 3.7-m SWIR imagery were exported to ArcGIS for manual evaluation and cleaning, wherein we assessed the model output and removed any obvious errors by hand to produce an estimate of rock garden distribution. This step required approximately 2 hours of analyst time. The resulting dataset contains a total of 0.76 km^2^ of rock gardens across Rapa Nui (Fig. 1).

**Fig. 4.**
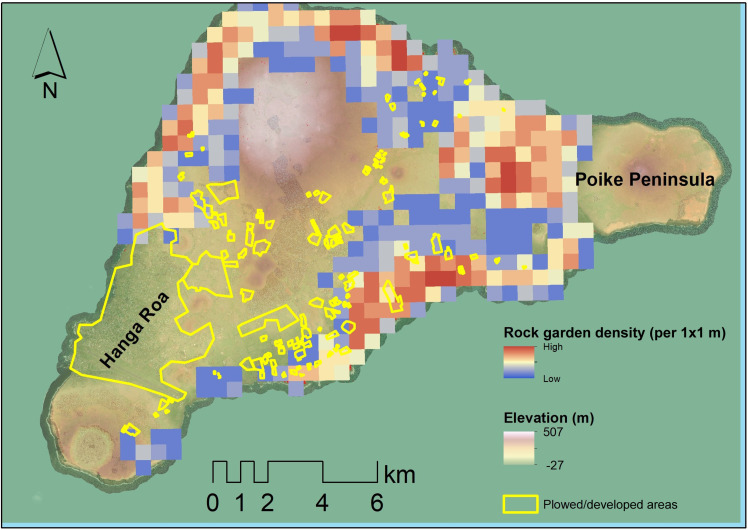
Map of urbanized and agriculturally disturbed areas in relation to calculated rock garden density.

In assessing the extent of rock gardening destruction that urbanization and modern agriculture may have caused, we estimate that ~25 km^2^ of the island has been affected. This estimate excludes the Poike Peninsula on the island’s eastern side. While there is some evidence of cultivation on Poike Peninsula, the area displays no evidence of rock gardening activities and is also excluded from previous analyses of rock garden distribution [see (*26*)]. Of this ~25 km^2^, >5 km^2^ of the cultivated areas are in zones where no rock gardens have been discovered during ground investigations or remote sensing surveys (see Fig. 4). Most (~17 km^2^) fall within or adjacent to Hanga Roa, the principal modern settlement on Rapa Nui.

## DISCUSSION

Our results show that SWIR offers an effective means for more accurately identifying lithic cultivation practices across Rapa Nui’s landscape. Our estimates offer greater overall accuracy and reliability than earlier estimates (see Tables 1 and 3; also see "Data and materials availability"). Ladefoged *et al.*’s (*26*) mulching estimates ranged from 4.3 to 21.1 km^2^. Our study estimates indicate that the total area of rock gardening is 0.76 km^2^, approximately one-fifth of the most conservative previous estimates, including many misclassified colluvium areas, lava flows, and roadways. Since rock gardening was significantly less prevalent than previously assumed, the island’s carrying capacity would also be substantially less than previous claims, even under optimistic assumptions of high productivity.

This study also has important implications for the utility of remote sensing in archaeology more broadly. Many instances remain where detecting archaeological landscapes is difficult using traditional multispectral datasets (VNIR). Detecting subtle changes to vegetative health, moisture retention properties of soils and plants, and even differentiating between soils and soil composition are crucial (*39*–*43*). This case study demonstrates that SWIR data can distinguish archaeological landscape components that often blend in with their surroundings (like rock gardens) from their surroundings where other components of the electromagnetic spectrum do not have enough spectral discriminatory power to make accurate identifications. As technology advances, the spectral resolution offered by the SWIR spectrum will open important doors for archaeological discovery in many regions around the world, particularly in rocky, volcanic landscapes. While VNIR is useful for various tasks, high-resolution SWIR provides the spectral resolution needed to distinguish anthropogenic landscape components of materials similar to natural bedrock and outcrop features.

### Reevaluating population estimates for Rapa Nui’s rock gardening infrastructure

Our results call for a reexamination of recent work by Puleston *et al.* (*6*) that uses previous estimates of rock gardening to model population sizes. One of the dominant staple crops frequently grown in rock gardens on Rapa Nui was sweet potato (*Ipomoea batatas*) (*11*, *23*). Puleston *et al.* (*6*) rely on estimates of sweet potato yields to approximate the pre-European contact population size of Rapa Nui and report that 1 ton/year of sweet potato produces a yield of 2809 kcal/day. Using their reported values for caloric requirements for the population (maximum of 2785 kcal per day per person) and their calculated yields of sweet potato (tons per hectare per year), they conclude that population sizes on Rapa Nui could have exceeded 16,000 [Table 3; also see (*6*)]. In contrast, baseline estimates of the population size using our new rock gardening data suggest that population sizes could not have exceeded 4000 (Table 3). It is essential to clarify that these estimates are based solely on sweet potatoes and refer to the population that could, theoretically, have been supported by rock gardening infrastructure alone. However, additional sources of subsistence (like marine foods and other terrestrial crops like bananas, yams, taro, and sugar cane) would have increased the island’s carrying capacity.

**Table 3.** Estimates of sweet potato yields and population sizes following mulching estimates of (*6*, *26*) and this study. Estimates of sweet potato yields and population sizes by cultivation frequency and soil nitrogen (N) availability following the methods of Puleston *et al.* (*6*) (Table 1) and our revised estimates for rock garden areas. We use the same ratios as Puleston *et al.* (*6*) (100% for continuous, 50% for shifting 5 years off 5 years on, and 25% for shifting 15 years off 3 years on). Sweet potato yields (tons/ha/year) were calculated by Puleston *et al.* (*6*).

Cultivation frequency	N availability	Sweet potato yield (tons/ha/year)	Rock garden area estimate	Land available for rock gardening (ha)	Sweet potato yield (kcal/day)	Number of individuals supported
Continuous	Low	1.46	(*26*)	3,133.9	12,852,562.6	4,614
Continuous	High	5.09	(*26*)	3,133.9	44,807,906.8	16,089
Shifting (5/5)	Low	2.38	(*26*)	1,566.9	10,475,384.6	3,761
Shifting (5/5)	High	8.00	(*26*)	1,566.9	35,211,376.8	12,643
Shifting (15/3)	Low	5.61	(*26*)	522.3	8,230,659.3	2,955
Shifting (15/3)	High	17.60	(*26*)	522.3	25,821,676.3	9,271
Continuous	Low	1.46	This study	760	3,116,886.4	1,119
Continuous	High	5.09	This study	760	10,866,335.6	3,901
Shifting (5/5)	Low	2.38	This study	380	2,540,459.6	912
Shifting (5/5)	High	8.00	This study	380	8,539,360.0	3,066
Shifting (15/3)	Low	5.61	This study	126.67	1,996,075.4	716
Shifting (15/3)	High	17.60	This study	126.67	6,262,384.6	2,248

To explore the implications of additional food sources on population estimates, we need to account from nonterrestrial sources and terrestrial foods grown in areas outside of rock gardening structures. Dietary estimates suggest that ~35 to 45% of the Rapanui diet was from marine sources (*24*). Because of low soil quality and other climatic considerations, additional crops like banana, taro, and sugar cane were not optimal crops for maximizing caloric returns but did serve as a supplement to the Rapanui diet (*11*, *25*). Thus, the impact of these crops on population sizes is likely to have been small, introducing dietary variety but limited increase to carrying capacity. The average carrying capacity of rock gardening calculated in this study is ~2000. If we add 50% to this estimate to account for marine and additional terrestrial foods from nonmulched areas, we arrive at an estimated carrying capacity of ~3000 people. This value matches historical accounts of population size at the time of European arrival in the 18th century (*44*, *45*). However, in addition to the impacts of crops grown outside of rock gardening and mulched areas, varied annual and seasonal growing conditions and changes to the amount of land used for cultivation over time are important considerations when estimating carrying capacity. Thus, further work is needed to model pre-European contact population sizes on Rapa Nui comprehensively and goes beyond this study’s scope.

Nevertheless, the arguments made by previous studies [e.g., (*6*)] rely on estimates of rock gardening features to model pre-European contact population sizes based on sweet potato cultivation. Our revised rock gardening estimates use the same logic but conclude that the range of possible population sizes supported by rock gardening infrastructure on Rapa Nui is significantly lower than previous estimates. Our estimates suggest that the maximum population supported by rock gardening is not ~17,000 as claimed through Ladefoged *et al.*’s (*26*) rock gardening calculations but just 3901 using our measurements.

**Fig. 5.**
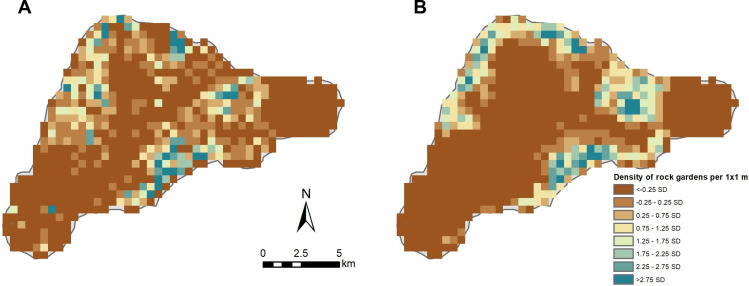
Comparison of rock gardening density distribution of Ladefoged *et al.* (*26*) and this study. (**A**) Minimal estimates from Ladefoged et al. (*26*). (**B**) Estimates from this study.

Overall, our results are more likely to underestimate small gardens that cannot be seen in the 3.7-m SWIR imagery but are unlikely to have missed other rock gardening features. When examining MaxEnt results for rock gardening classes, specifically, the model attained a true positive rate of over 78% and a true negative rate of over 96%, meaning that there is a low rate of error both for excluding real rock gardening features and from misidentifying other landscape components as rock gardens (see Supplemental Code). As such, our estimates likely come close to the total number of extant rock gardening features on Rapa Nui. The density of rock gardens was compared to Ladefoged and colleagues’ (*26*) most conservative estimates (minimal classification) by conducting a kernel density test of mulch locations using a 500 m × 500 m cell size [following (*26*)]. Kernel density calculates the density of features within a specified window (i.e., 500 m^2^). The overall location and density of gardens are similar to those of earlier studies, primarily clustered along coastal regions and largely absent further inland at higher altitudes (Fig. 5) (*4*, *26*). Previous island-wide estimates of rock gardening distribution contain numerous examples of false positives and negatives that were not replicated in this study using SWIR (Fig. 3). Additionally, the urban part of the island could have had rock mulch that has since been destroyed.

Such preservation issues present substantial challenges to archaeologists, and this study is no exception. Using modern (2015 to 2017) satellite observations means that features that have been destroyed are no longer identifiable using these approaches alone, and ground-based analysis is required to verify further or to challenge our conclusions. On the basis of our assessment of surface disturbances, there is little doubt that some rock gardening infrastructure has been destroyed. Still, we suspect that the destruction is limited as most developed areas would not have had rock gardening infrastructure due to their location on the island (Fig. 4). Most rock gardens are clustered near the coast, and over 5 km^2^ of the cultivated areas are in zones where no rock gardens have been discovered during ground investigations or remote sensing surveys.

Nonetheless, our results require careful interpretation, as the total amount of rock gardening features may have been higher than what is reported but have since been destroyed. Furthermore, it is important to emphasize that not all of the rock gardens identified here were necessarily contemporaneous; some of these fields may be older than others, and the total amount of rock gardening infrastructure may have been significantly different or was more limited in extent at different points in the island’s history. Prior estimates of rock gardening infrastructure relied on satellite imagery from 2010 (*26*), and as such, our results are comparable despite the possible bias caused by the destruction of archaeological features. Furthermore, previous estimates of rock gardening excluded 45 km^2^ of land area from analysis due to cloud coverage and urban and agricultural infrastructure. Therefore, we investigated a greater proportion of the island’s land mass (due to SWIR’s ability to penetrate cloud cover) and greatly improved the accuracy of rock gardening estimates.

Ultimately, the absolute population size on Rapa Nui before European contact is currently unknown, as additional sources of food from nonmulched cultivation areas and other crops like banana and taro would have affected the overall carrying capacity. To accomplish this task, more extensive studies of contemporary cultivation areas could be carried out to determine how long certain areas have been used for food production. Additionally, the calculation of caloric returns from other staple crops on Rapa Nui would enhance estimates of likely food production and population sizes that could have been supported by these efforts. While a comprehensive evaluation of past population sizes goes beyond the scope of this investigation, our results will help to contribute to this area of ongoing research.

### Revisiting the Malthusian narrative and lessons for sustainability

Our estimates of rock garden prevalence on Rapa Nui are essential not only in the context of the ongoing debate about population size but also in the overall concept of “collapse” and “ecocide” on the island (*46*–*53*). Malthusian assumptions are prevalent in a wide range of studies that point to the pre-European contact population size of the island, drawing on previous rock gardening estimates that inflate the land area used for cultivation (*6*, *46*, *49*, *54*). Recent research has attempted to estimate the pre-European population size on the island using previous evaluations of lithic mulching, with estimates varying widely from ~3000 to more than 17,000 people (*6*). While an argument for a population size of 17,000 is problematic (*5*, *55*), the overestimation of lithic mulching, as demonstrated by our new estimates, warrants revisiting environmental carrying capacity and population estimates for Rapa Nui (Table 3).

Despite recent archaeological literature debunking ideas about Malthusian population overshoot, the premise that Rapanui society caused its own demise from unsustainable resource use and uncontrolled population increases has been widely popularized. While many researchers working on the island have shifted their narratives away from the assumptions of a pre-European collapse, the story remains prominent in disciplines such as ecology, paleoecology, and mathematics (*49*, *54*). Archaeology has much to offer other disciplines focused on contemporary conservation and resource management issues, but only when the archaeological record is accurately measured and interpreted [see (*56*)].

Our results add to a growing body of empirical research showing that Rapa Nui represents a prime example of how an isolated population with limited natural resources created a sustainable subsistence system, maintaining their numbers within the limitations of environmental carrying capacity (*55*). Contrary to popularized narratives about a runaway population size that overexploited natural resources (*37*, *46*), our results suggest that significant demographic increases (“overshoot”) did not occur in the past, given the limited extent of agricultural infrastructure. Using high-resolution SWIR imagery and machine learning, we detect rock gardens across Rapa Nui’s landscape with an accuracy of >80%. On Rapa Nui, our results help refine estimates of agricultural productivity, suggesting that previous estimates were between 5 and 20 times too high. This finding holds significant implications for estimates of population size and subsistence strategies of the Rapanui people before European contact. Future research will use the data generated here to model and estimate historical population sizes on the island comprehensively.

## MATERIALS AND METHODS

To generate an island-wide estimate for rock gardening distribution, we use a combination of high-resolution multispectral imagery from Worldview 3,archaeological survey data, and machine learning.

### High-resolution multispectral imagery

We obtained Worldview 3 imagery of the entire island with minimal cloud cover from DigitalGlobe (now Maxar) (Table 4). Images were acquired between July 2015 and July 2017. Data were delivered in 16-bit format for VNIR and 8-bit for SWIR. We converted the VNIR data to an 8-bit format to enhance processing capabilities and compatibility with the SWIR imagery.

**Table 4.** Worldview-3 imagery delivery specifications.

Image dates	Sensor	Wavelengths (μm)	Data processing
			Resolution: 3.70 m (SWIR),
	Coastal blue	0.400–0.450	1.2 m (VNIR), 0.3
	Blue	0.450–0.510	(panchromatic)
	Green	0.510–0.580	
	Yellow	0.585–0.625	Products: Orthos, mosaic
	Red	0.630–0.690	
2015-07-28	Red edge	0.705–0.745	
2015-09-09			Ortho control: rational polynomial coefficients (RPCs), Tie
2015-12-13			Points
2016-08-03	NIR1	0.770–0.850	
2016-08-03	NIR2	0.860–1.040	
2017-02-11			Ortho surface: SRTM30m
2017-07-22	SWIR-1	1.195–1.225	
2017-07-22	SWIR-2	1.550–1.590	Resampling: Cubic
2017-07-29	SWIR-3	1.640–1.680	
	SWIR-4	1.710–1.750	
	SWIR-5	2.145–2.185	Projection/datum: UTM
	SWIR-6	2.185–2.225	12Sth WGS84
	SWIR-7	2.235–2.285	
	SWIR-8	2.295–2.365	
			European Petroleum Survey Group (EPSG) code: 32712

SWIR imagery has not been extensively used in archaeological research despite its substantial potential, largely due to the historically low spatial resolution of commercial SWIR sensors [but see (*35*)]. While lower spatial resolution SWIR has been incorporated into a few landscape archaeological studies (*57*–*59*), its precise role and importance for archaeological prospection and feature identification have not been well explored.

With the launch of DigitalGlobe’s (now Maxar’s) Worldview-3 satellite in 2014, high-resolution SWIR became available for the first time in eight distinct bands (see Table 4). While data are captured in 3.7-m spatial resolution, images provided by Maxar were restricted to 7.5-m resolution for public use until 2019. Even with these lower resolutions, these data have proven useful in various studies (*60*–*62*) and even showed promise in an archaeological pilot study (*63*). Since 2019, however, we know of only one archaeological study that has made use of the SWIR data now available at the original spatial resolution of 3.7 m per pixel, demonstrating that SWIR can aid in detecting archaeological features in the Middle East (*39*).

Multispectral imagery records light reflected from the Earth’s surface in different wavelengths across the electromagnetic spectrum. Different landscape elements will reflect or absorb light in different ratios, and multispectral data can thus be used to classify land cover and specific environmental variables based on their geophysical profiles. Water, for example, is absorbed in the NIR (0.76 to 1.04 μm), but reflected in the blue (0.45 to 0.51 μm), while plants tend to absorb blue and red light but reflect more green (0.51 to 0.58 μm) and NIR light. The SWIR range (1.195 to 2.365 μm) is particularly useful for geologic applications because of its ability to distinguish between dry and wet soils, as water is absorbed in the SWIR spectrum, and their reflectance or absorption characteristics can distinguish different minerals.

Within the context of rock gardening on Rapa Nui, the use of SWIR data permits for a greater degree of discrimination between rock gardens, vegetation, natural rock outcrops, and bare soils due to its high sensitivity to moisture variations and mineral composition, as has been demonstrated by a variety of lithological and agricultural studies [e.g., (*28*, *64*, *65*)]. For landscape archaeological investigations, SWIR can aid in identifying rock and soil-based features that may otherwise blend into the surrounding landscape when viewed through other types of multispectral imagery.

### Training data collection

To train machine learning classifiers to recognize lithic mulch, we compiled a training dataset consisting of known lithic mulch and other surrounding environmental contexts recorded during ground surveys conducted in 2019 and 2023 (Table 5). For this analysis, all lithic mulching and rock gardening forms are grouped into a single land-cover class. The training samples contain data points throughout the island to ensure that we captured a wide range of land-cover variability. We then exported these data as shapefiles and analyzed them in R (*66*).

**Table 5.** Training dataset used for machine learning.

Class	Number of features	Total area (km^2^)
Bare soil	27	0.101
Bedrock/colluvium	66	0.090
Forest/trees	25	0.235
Grass	36	0.090
Mulch	40	0.031
Urban/developed	30	0.053

### Machine learning classification

To identify lithic mulch gardens, we trained three machine learning algorithms: (i) maximum entropy (MaxEnt), (ii) random forest (RF), and (iii) maximum likelihood classification (MLC). These analyses were conducted in R v. 4.0.2 (*66*) using the RSToolbox, caret, MIAMaxEnt, and randomForest packages (*67*–*70*). All three algorithms are well established for landscape classification, and we specifically chose to test MaxEnt and RF algorithms because they are known to work well with incomplete datasets and smaller sample sizes. We assess MLC because it was previously used to identify rock gardening structures on Rapa Nui (*26*). We ran these algorithms on the VNIR and SWIR datasets to compare the ability of the two datasets to identify rock gardening features.

MaxEnt has been shown to produce highly accurate results while minimizing overfitting issues of some other machine learning models (*71*). MaxEnt works well with incomplete datasets by assuming that decisions have uniform distributions of choices based on training datasets, therefore making assumptions exclusively about known data and not requiring the absence of information (*72*, *73*). RF is a nonparametric method that classifies data using a bootstrapped decision tree approach and performs well with small amounts of training information (*74*). Decision trees operate using recursion to classify data into specific subsets. In RF, each decision tree reaches a conclusion, and the greatest number of trees in the agreement is selected as the “true” classification. Finally, MLC is a widely used parametric classification approach that is derived from Bayes’ theorem (*75*, *76*). It considers variance and covariance of data when making classification decisions but usually requires greater amounts of training data to work effectively (*77*). MLC can be used with non-normal datasets, but the more skewed the distribution, the poorer the algorithm performs (*78*). In their analysis of WorldView-2 imagery, Ladefoged *et al.* (*26*) used MLC to identify rock gardens.

For each machine learning model, we used an 80-20 split of our training samples for training and validation, respectively. We tuned each model (to alleviate overfitting or other performance issues) automatically using the “tune_length” parameter in the caret package in R (see supplemental code in "Data and materials availability"). We set the tune_length to 1 for all models. Our model tested other tune_lengths, but these values did not alter performance and increased computational time.

### Assessment of rock gardening distribution on Rapa Nui

Next, to determine whether combining SWIR with VNIR provided additional improvements to rock garden identification, we pan-sharpened the SWIR imagery to the same 1.2-m resolution as the VNIR data with a principal components analysis (PCA) method using the RSToolbox package in R (*66*, *68*). For our analysis, we excluded the area covered by the town of Hanga Roa, where modern developments have replaced the archaeological landscape, including rock garden areas. We use the same area as (*26*) in their analysis. We combined the pan-sharpened SWIR data with the VNIR images and applied the best-performing machine learning algorithm from our previous tests. We exported the best-performing classification results as raster files after evaluating the performance metrics from the combined, pan-sharpened imagery results with the separate SWIR and VNIR tests.

We then converted the classified raster to a shapefile using the “Raster to Polygon” tool in ArcMap 10.8.1. We manually checked the resulting polygon file of rock garden locations for errors. We removed features in areas influenced by cloud coverage and those produced by the shadow effect often occurring in modern urban areas. We checked for misclassified polygons in previously misidentified areas, such as roadways, soil patches, and natural lava flows. Additionally, we removed all polygons less than 10 m^2^ in total area. Most polygons of this size were artifacts introduced from the raster-polygon conversion and represent single pixels that do not align with garden features, given the resolution of the available data. While some of these 10-m^2^ polygons might represent small garden features, we removed them because they are not visible in 3.7-m imagery.

### Assessing potential rock gardening infrastructure destroyed by modern land use

One limitation of using modern remote sensing observations to identify ancient structures is that modern agricultural activities, urbanization efforts, and environmental factors like erosion can destroy or damage surface deposits. To account for the possibility that some ancient rock gardening is missing from our final results, we manually analyzed the VNIR Worldview-3 imagery (with 1.2-m resolution) to identify obvious signs of surface disturbance (i.e., plow marks, field boundaries, urban development). We digitized polygons around plowed field boundaries and urbanized areas and quantified the land area affected by these modern land-use practices.
